# P-559. Cohort of Individuals Bitten by a Tick and Prospective Evaluation of Post-Exposure Prophylaxis for Lyme Disease in Quebec (BQTick): a Pilot Study

**DOI:** 10.1093/ofid/ofaf695.774

**Published:** 2026-01-11

**Authors:** Lorraine De Grâce, Jérôme Pelletier, Jeanne Tremblay, Véronique Noël, Alex Carignan

**Affiliations:** Université de Sherbrooke, Sherbrooke, QC, Canada; Université de Sherbooke, Sherbrooke, Quebec, Canada; Université de Sherbrooke, Sherbrooke, QC, Canada; Centre de recherche du CHUS (CRCHUS), Sherbrooke, Quebec, Canada; Universite de Sherbrooke, Sherbrooke, QC, Canada

## Abstract

**Background:**

The BQTick project aims to establish a biobank designed to enable real-time identification of emerging tick-borne pathogens in Quebec, Canada, and monitor the effectiveness of post-exposure prophylaxis (PEP) for Lyme disease following a tick bite. We assessed the feasibility of conducting a prospective follow-up of individuals bitten by *Ixodes scapularis* ticks in the Estrie region, with the aim of guiding the development of a prospective cohort study.

**Figure:**
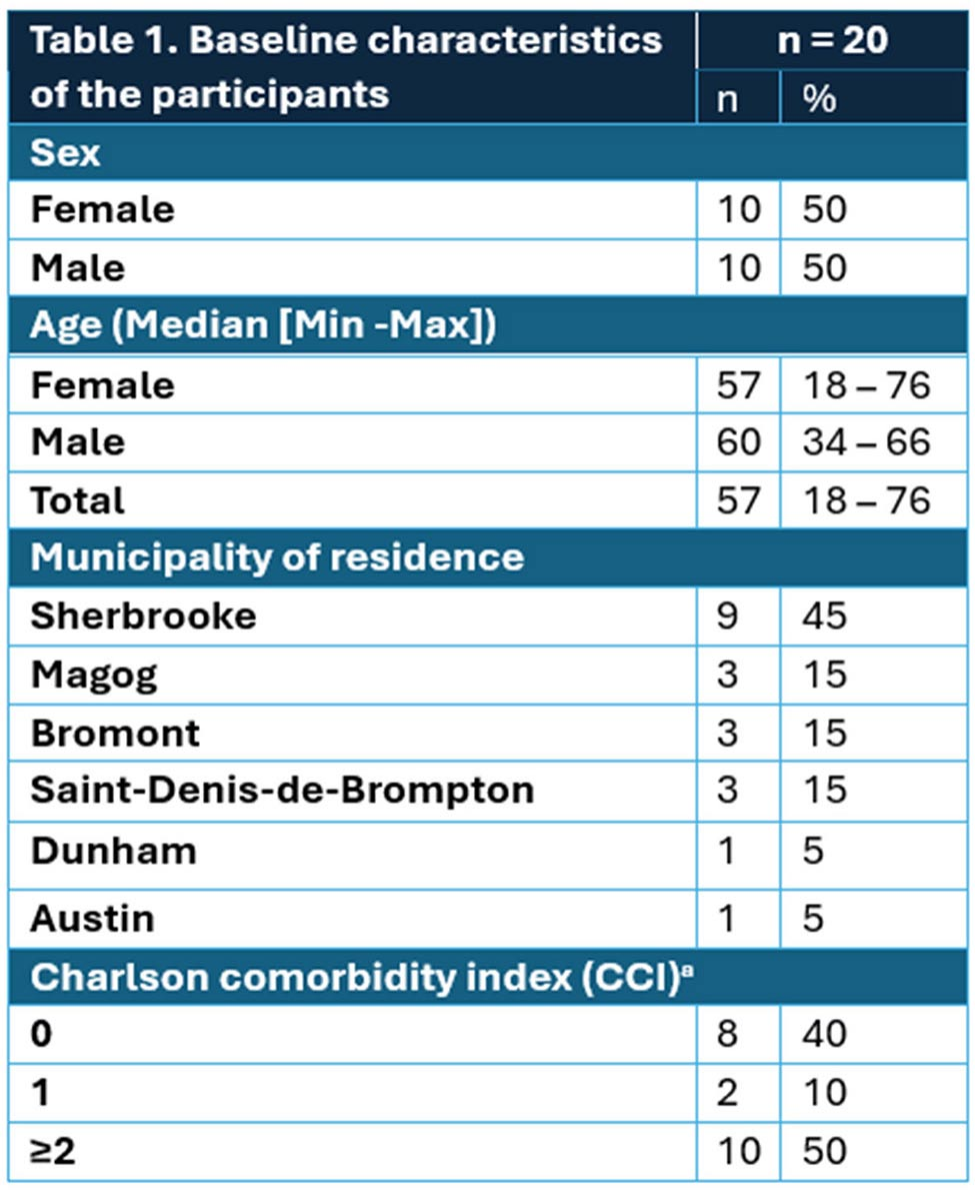

**Figure:**
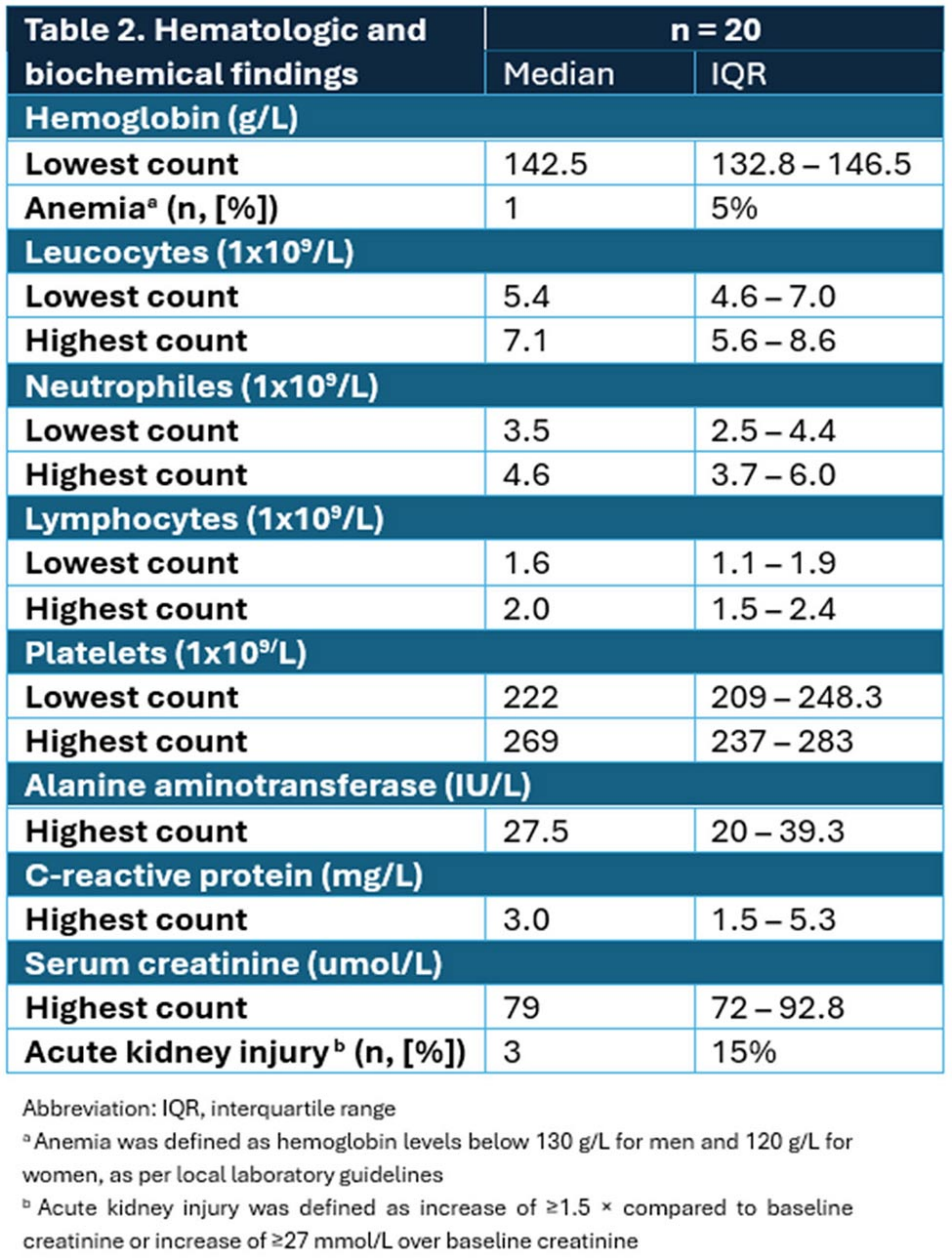

**Methods:**

For this pilot study, we recruited 20 participants meeting the following criteria: i) have been bitten by an *Ixodes scapularis* tick within the past 7 days at the time of visit and ii) being able to provide the tick. Four follow-up visits were conducted at 7-, 14-, 30-, and 90-days post–tick bite. The ticks recovered from participants were stored at –80 °C until shipment for laboratory analysis to identify tickborne pathogens. During each visit, standardized questionnaires were administered to collect sociodemographic, clinical, and paraclinical data. Microbiological testing included serology for Lyme disease, anaplasmosis, and babesiosis, as well as a PCR testing for each of these infections and a peripheral blood smear for the detection of morulae.

**Figure:**
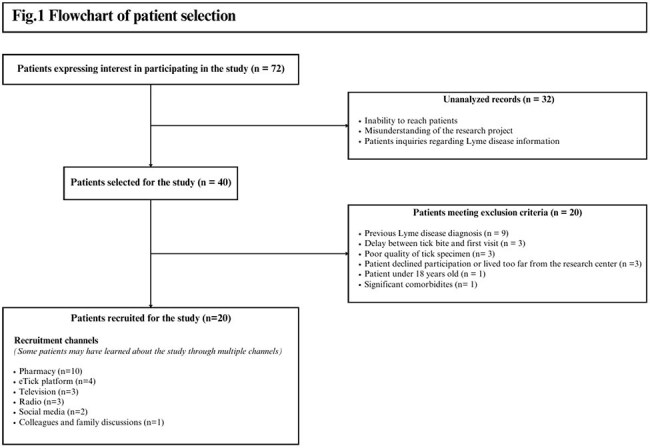

**Results:**

Overall, 20 participants were enrolled. The patients’ demographic and clinical characteristics are summarized in Table 1 and the hematologic and biochemical laboratory findings are listed in Table 2. Most patients were referred by a pharmacist (10/20; 50%) and 15/20 patients received PEP prior to their enrolment visit. Initial visit occurred at a median of 4.5 days (interquartile range 3.8 – 6.0 days) following tick bites. Of 72 interested patients, 27.8% were retained for the study. (Figure 1). All patients completed the 90-day follow-up, and 19/20 patients (95%) completed all scheduled visits. A total of 79 out of the 80 visits initially planned in the protocol were completed (98.5%). All patients remained asymptomatic and none of them tested positive for *B. burgdorferi and. A. phagocytophilum. Babesia* spp. results are pending.

**Conclusion:**

This pilot study suggests that creating a provincial biobank through a prospective cohort of tick-bitten patients is feasible. An increase in the share of patients without PEP would allow a better assessment of its effectiveness.

**Disclosures:**

Alex Carignan, MD, MSc, GSK: Advisor/Consultant|GSK: Grant/Research Support|GSK: Honoraria|Innomed Pharma: Advisor/Consultant|Merck: Advisor/Consultant|Moderna: Advisor/Consultant|Moderna: Honoraria|Palladin Labs: Advisor/Consultant|Pfizer: Advisor/Consultant|Pfizer: Grant/Research Support|Sandoz: Honoraria

